# Diagnostic performance, molecular analysis, and complications in CT-guided percutaneous biopsies of lung nodules with 20-gauge needles

**DOI:** 10.36416/1806-3756/e20250158

**Published:** 2025-12-11

**Authors:** Priscila Mina Falsarella, Andre Dubinco, Marcelo da Rosa Bortot, Paulo Vidal Campregher, Renée Zon Filippi, Antonio Rahal, Rodrigo Gobbo Garcia

**Affiliations:** 1. Centro de Medicina Intervencionista, Hospital Israelita Albert Einstein, São Paulo, SP, Brasil.; 2. Laboratório de Genética Molecular, Hospital Israelita Albert Einstein, São Paulo, SP, Brasil.; 3. Departamento de Anatomia Patológica, Hospital Israelita Albert Einstein, São Paulo, SP, Brasil.

## TO THE EDITOR,

Computed tomography (CT)-guided percutaneous biopsy of lung nodules is a minimally invasive procedure that has become the modality of choice for obtaining target lung tissue for histopathological and molecular analyses, owing to its high diagnostic accuracy (64-97%) and low rates of serious complications.^1^

Complications following this procedure are uncommon, typically mild, and usually do not require invasive interventions.^2^ Pneumothorax is the most frequent complication, occurring in 8-64% of cases. It is generally laminar, stable, and asymptomatic. Studies have shown a higher risk in patients over 60 years of age and in biopsies performed with large-gauge needles (< 18-gauge).^3^ Bleeding is a potentially serious complication that requires close monitoring during the periprocedural period. Risk factors include puncture of lesions smaller than 2.0 cm and the use of large-gauge needles.^1^ Other possible complications include air embolism, tumor seeding along the needle tract, and death.^3^

In the era of personalized medicine, histopathological and immunohistochemical analyses alone are often insufficient to guide optimal therapeutic strategies. In this context, the identification of genetic alterations amenable to targeted therapies in lung adenocarcinoma has led to a significant expansion in treatment options, allowing for increasingly individualized therapeutic decisions based on the tumor’s molecular profile. These molecular characteristics include mutations in genes such as epidermal growth factor receptor (*EGFR*), *KRAS*, *HER2*, and *BRAF*; rearrangements involving *ALK*, *ROS1*, *NTRK1-3*, and *RET*; and alterations in *MET*. Approximately 60-80% of non-small cell lung cancers harbor genomic alterations that are eligible for targeted therapy. In addition, these tumors may express cell surface proteins such as PD-L1, either alone or in combination with other biomarkers, making patients potential candidates for immunotherapy.^4^ ^,^ ^5^

Jamshidi et al. (2017) demonstrated that using an 18-gauge biopsy needle with one to four passes yielded 1,310 to 3,907 ng of genetic material. In contrast, using a 20-gauge needle with the same number of passes yielded only 330 to 984 ng, indicating that the 18-gauge needle provided a 4.8- to 5.7-fold greater yield than the 20-gauge needle.^6^ When comparing two passes versus a single pass, the yield was 2.4 to 2.8 times greater with 18-gauge needles than with 20-gauge needles.^6^ To optimize nucleic acid yield for personalized approaches, the use of smaller-gauge needles with fewer passes is preferable to larger-gauge needles with more passes.^6^ Additionally, Silk et al. (2018) demonstrated that 200 to 250 ng of DNA is generally sufficient for molecular analysis using most commercially available kits, without the need to repeat the extraction process.^7^

The aim of the present study was to evaluate the diagnostic performance, complication rate, and adequacy of genetic material obtained from target lesions for molecular analysis using CT-guided percutaneous biopsy of pulmonary nodules, performed with a semi-automatic coaxial cutting system with a 19-gauge introducer and a 20-gauge biopsy needle.

This retrospective, single-center cohort study was conducted at a quaternary care hospital following approval by the institutional review board. Between January 2017 and February 2018, 97 consecutive cases of patients who underwent CT-guided biopsy of lung nodules/masses due to clinical suspicion of malignancy were analyzed in the Center of Interventional Medicine. 

All procedures were performed under sedation or general anesthesia. All patients underwent a non-contrast helical computed tomography (CT) scan to confirm the size and location of the nodule and to plan the access route.

Tissue samples were obtained using a 19/20-gauge coaxial biopsy needle system (Bard Mission, Bard, USA). With the needle positioned within the nodule, one to three fragments were collected. An on-site pathologist assessed sample adequacy using the imprint cytology technique to determine whether the material was representative. Following this initial assessment, the specimens were immediately fixed in 10% formaldehyde solution. At the end of the procedure, a blood patch was injected along the coaxial needle tract to reduce the risk of pneumothorax, followed by a non-contrast helical CT scan to evaluate for possible immediate complications. An anteroposterior chest X-ray was performed 1 hour after the procedure to assess for delayed complications. Patients were monitored for 4 hours post-procedure before discharge from the anesthesia care unit.

Of the total samples, 22 were submitted for genetic analysis (Table 1). In the OncoScreen test, DNA and RNA are extracted simultaneously. When the sample is small, DNA extraction may be compromised, requiring an alternative technique; in such cases, the result was classified as suboptimal. For the FoundationOne test, results were considered suboptimal when the sample did not meet all quality criteria (e.g., inadequate quantity or quality), but analysis was still feasible. Samples with either optimal or suboptimal results were considered adequate for the purposes of this study.

**Table 1:** Description of pulmonary nodules: test type, nodule size, number of fragments, and test accuracy.

	OncoScreen	FoundationOne
Sample size	14	8
Size (median), cm	2.55 (0.7 - 8.8)	1.9 (1.2 - 8.0)
Number of fragments (median)	8	8.5
Test accuracy		
Adequate	12	4
Suboptimal	1	4
Insufficient	1	0

**Figure 1:**
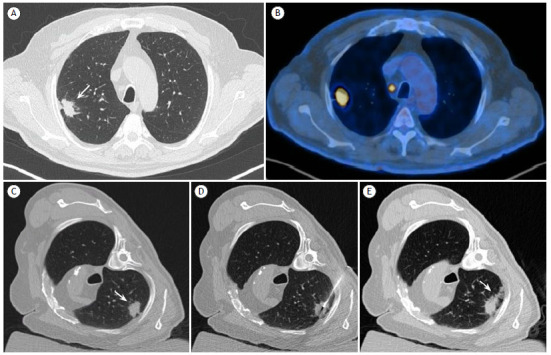
(A) Non-contrast chest computed tomography (CT) showing a non-calcified pulmonary nodule with spiculated margins (white arrow) located in the peripheral region of the posterior segment of the right upper lobe. The lesion measures approximately 2.3 × 1.2 cm in its largest axial dimensions and is adjacent to the parietal pleura, suggestive of primary lung neoplasia. (B) PET-CT with 18F-FDG showing intense glycolytic activity in the pulmonary nodule (SUVmax: 12.5), in addition to marked radiotracer uptake in a mediastinal lymph node in the right paratracheal chain (SUVmax: 10.8), suggestive of secondary involvement. (C) Chest CT during pre-procedural planning, with the patient in the right oblique lateral decubitus position. (D) Intra-procedural chest CT showing placement of the 19-gauge coaxial cannula via posterior access. (E) Post-procedural control chest CT showing the blood patch along the path of the coaxial cannula (white arrow).

Among the 22 cases analyzed, 14 patients underwent the OncoScreen test and 8 underwent the FoundationOne test. In the OncoScreen group, 12 samples were considered adequate, 1 was suboptimal, and 1 was insufficient. As for the patients who underwent the FoundationOne test, 4 samples were deemed adequate and 4 were suboptimal (Table 1; Figure 1).

The mean number of fragments obtained during biopsy was 8.2 (median = 8) in patients who underwent genetic analysis, compared to 6.9 (median = 7) in those who did not. In the OncoScreen group, the mean number of fragments was 8.55 (median = 8) in patients with adequate samples and 9 (median = 9) in the suboptimal case. In the FoundationOne group, the mean was 8.5 fragments (median = 8.5) in cases with adequate material and 6.75 (median = 7) in those with suboptimal material.

Among the complications observed in the patients who underwent genetic analysis, there was 1 case (4.5%) of pneumothorax requiring chest drainage and 1 case (4.5%) of hemothorax that did not require intervention. In the group without additional material sent for genetic testing, 6 cases (8.5%) of pneumothorax requiring chest drainage were observed, with no instances of hemothorax.

Histopathological and immunohistochemical studies may be insufficient, making molecular analysis essential for identifying driver mutations and other biomarkers that enable targeted treatment.^2^ ^,^ ^8^

Adequate sampling of the target lesion is crucial for accurate diagnosis and for obtaining tissue with preserved architecture, sufficient cellularity, and intact genetic material after fixation, ensuring that genetic extraction is viable for the analysis of potential driver mutations.^9^ Studies suggest that at least 100 tumor cells are recommended for reliable molecular analysis, while *in situ* hybridization (FISH) requires a minimum of 50 cell nuclei.^9^

However, the minimum number of fragments and the optimal needle gauge for adequate genetic testing performance have not yet been well established. In a study by Hoang et al. (2018), lung biopsies performed with 20-gauge needles yielded adequate material in 69% of cases when an average of 5 fragments was obtained, and in 92% of cases with an average of 10 fragments. In an *ex vivo* animal model, the same study concluded that 16- and 18-gauge needles required fewer fragments to achieve comparable diagnostic yield.^10^

In the present study, adequate material was obtained in 95.4% of cases (21/22) using 20-gauge needles with an average of 8.2 fragments, demonstrating good performance. Notably, there was no increase in post-procedural complications among patients who underwent genetic analysis (9%) compared to those who did not have additional fragments collected for this purpose (8.5%).

This study had some limitations. First, it was a retrospective analysis. Second, only a small number of patients underwent genetic analysis of the material. 

In conclusion, the use of a 19/20-gauge coaxial biopsy needle system for pulmonary nodule biopsy requiring genetic analysis proved effective, demonstrating a high diagnostic yield without a significant increase in procedure-related complications when compared to patients who did not undergo genetic testing.
